# Compound Heterozygous *RYR1* Variants in a Patient with Severe Congenital Myopathy: Case Report and Comparison with Additional Cases of Recessive *RYR1*-Related Myopathy

**DOI:** 10.3390/ijms251910867

**Published:** 2024-10-09

**Authors:** Sören Janßen, Leoni S. Erbe, Moritz Kneifel, Matthias Vorgerd, Kristina Döring, Krzysztof P. Lubieniecki, Joanna M. Lubieniecka, Wanda M. Gerding, Nicolas Casadei, Anne-Katrin Güttsches, Christoph Heyer, Thomas Lücke, Hoa Huu Phuc Nguyen, Cornelia Köhler, Sabine Hoffjan

**Affiliations:** 1 Department of Neuropediatrics, University Children’s Hospital, Ruhr-University Bochum, 44791 Bochum, Germany; soeren.janssen@rub.de (S.J.); thomas.luecke@rub.de (T.L.); cornelia.koehler@kklbo.de (C.K.); 2 Department of Human Genetics, Ruhr-University Bochum, 44801 Bochum, Germany; leoni.erbe@rub.de (L.S.E.); kristina.doering@rub.de (K.D.); krzysztof.lubieniecki@ruhr-uni-bochum.de (K.P.L.); joanna.lubieniecka@rub.de (J.M.L.); wanda.gerding@rub.de (W.M.G.); huu.nguyen-r7w@rub.de (H.H.P.N.); 3 Department of Neurology, Heimer Institute for Muscle Research, University Hospital Bergmannsheil, Ruhr-University Bochum, 44789 Bochum, Germany; moritz.kneifel@rub.de (M.K.); matthias.vorgerd@bergmannsheil.de (M.V.);; 4 Institute of Medical Genetics and Applied Genomics, University Tübingen, 72074 Tübingen, Germany; nicolas.casadei@med.uni-tuebingen.de; 5 NGS Competence Center Tübingen, 72076 Tübingen, Germany; 6 Institute for Pediatric Radiology, Katholisches Klinikum Bochum, Ruhr-University Bochum, 44791 Bochum, Germany; christoph.heyer@rub.de; 7 Center for Rare Diseases Ruhr (CeSER), 44791 Bochum, Germany

**Keywords:** ryanodine receptor 1 (RYR1), whole genome sequencing (WGS), congenital myopathy, splice variant, *RYR1*-related myopathies

## Abstract

Pathogenic variants in the ryanodine receptor 1 (*RYR1*) gene are causative for a wide spectrum of muscular phenotypes, ranging from malignant hyperthermia over mild, non-progressive to severe congenital myopathy. Both autosomal dominant and recessive inheritance can occur, with the more severe forms usually showing recessive inheritance. However, genotype–phenotype correlations are complicated due to the large size of the gene and heterogeneous phenotypes. We present a 6-year-old patient with severe congenital myopathy, carrying a heterozygous pathogenic *RYR1* variant inherited from the healthy mother. Through whole genome sequencing we identified a second, deep intronic *RYR1* variant that has recently been described in another patient with severe congenital myopathy and shown to affect splicing. Segregation analyses confirmed the variants to be compound heterozygous. We compared our patient’s phenotype to that of the patient from the literature as well as five additional patients with compound heterozygous *RYR1* variants from our center. The main overlapping features comprised congenital onset, predominant muscular hypotonia, and normal creatine kinase (CK) levels, while overall clinical expression varied substantially. Interestingly, both patients carrying the new intronic splice variant showed a very severe disease course. More widespread use of genome sequencing will open the way for better genotype–phenotype correlations.

## 1. Introduction

The *RYR1* gene located at chromosome 19q13.2 encodes for the ryanodine receptor, a calcium release channel in the skeletal muscle sarcoplasmic reticulum. The gene is approximately 160 kb in size and comprises 106 exons. In cohort studies, *RYR1* variations account for 33 to 59% of patients with congenital myopathies, thus constituting one of the most frequent causes within this group of disorders [1]. However, *RYR1*-related myopathies comprise a highly heterogeneous group of muscular disorders, ranging from malignant hyperthermia (MH) without clinically evident muscular disease to severe congenital muscular myopathy, sometimes even fatal [2]. Since the detection of the *RYR1* gene in 1988, a large range of clinical and histopathological phenotypes have been linked to this gene. These were summarized in historical order in Lawal et al. [3] and include MH, central core disease (CCD), centronuclear myopathy (CNM), multiminicore disease (MmD), and congenital fiber-type disproportion (CFTD). Both autosomal recessive and autosomal dominant inheritance can occur. MH and CCD typically show dominant inheritance, while CNM, MmD, and CFTD are rather associated with a recessive mode of inheritance. However, it became evident that the histopathological features in the muscle biopsy (such as central cores or multiminicores), originally used for classification, were not specific to certain phenotypes and could change over the course of time [3]. Therefore, the term “*RYR1*-related myopathies” was introduced instead, and some authors even suggested the term “*RYR1*-related disorders” to best cover the broad spectrum [3]. The *RYR1* gene is one of the largest genes in the human genome, and numerous variants have been described of this gene. Interpretation of the clinical relevance of these variants can be challenging. Autosomal recessive inheritance is usually associated with more severe symptoms [4], but more precise genotype–phenotype correlations are still pending. Clinical severity can vary substantially, even within the dominant or recessive phenotypes.

Whole exome sequencing (WES) has been routinely used for neuromuscular disorders over recent years. However, this technique cannot identify deep intronic and structural variation, and diagnostic yield for myopathies has been reported between 46% and 65% [5,6]. We present here a 6-year-old German patient with a severe congenital myopathy who was known to carry one pathogenic, maternally inherited, and presumably recessive *RYR1* variant since his first year of life. Whole genome sequencing (WGS) detected a second *RYR1* variant in intron 69. Segregation analysis revealed that the variant is paternally inherited. Furthermore, this variant has recently been described in another patient and shown to affect splicing. Thus, we describe a second patient in whom WGS led to the definitive diagnosis of an *RYR1*-related congenital myopathy. We compare the phenotype of our patient with the phenotype of the published patient, as well as five other patients who were determined to be compound heterozygous for *RYR1* variants in our center over the last five years.

## 2. Results

### 2.1. Case Report

**Figure 1:**
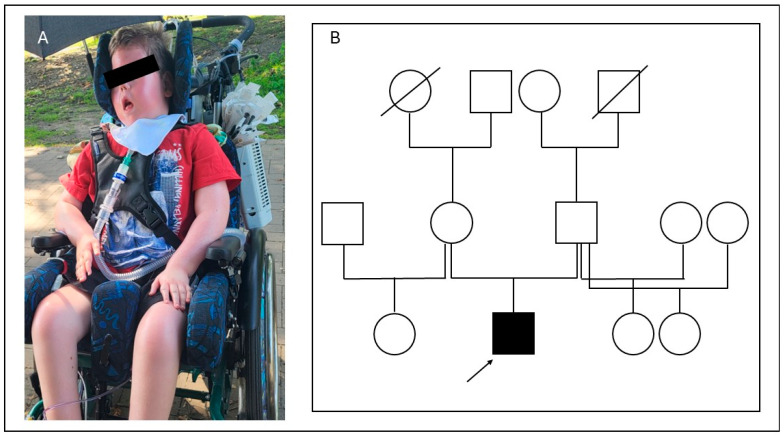
(**A**): The patient at the current age of 6 years, wheelchair-bound and with constant ventilation. (**B**): Pedigree of the patient (squares: male family members, circles: female members; crossed symbols: deceased family members; the affected individual is shown with a solid symbol and marked with an arrow).

The male patient is 6 years old at the present time. He was born to healthy, non-consanguineous German parents; the family history was uneventful regarding muscular diseases (Figure 1). During pregnancy, the fetus showed reduced movements and breech presentation. In addition, gestational diabetes and preeclampsia were diagnosed, due to which a C-section was performed at gestational week 34 + 6. Congenitally, the patient had generalized symmetric hypotension and required ventilation. Since birth, he has shown severe global muscle weakness, which also affects facial and pharyngeal muscles. The patient requires continuous ventilation. In addition, a bilateral dislocation of the hip and scoliosis were diagnosed. His speech comprehension seems to be unremarkable. He communicates by vocalizations and nodding. He is also able to move fingers and feet minimally and to open his eyes. Cardiological examination did not reveal any abnormalities. An MRI of the brain and the examination of nerve conduction velocities were also normal. There were no organ malformations, and the creatine kinase (CK) value was within the normal range.

**Figure 2:**
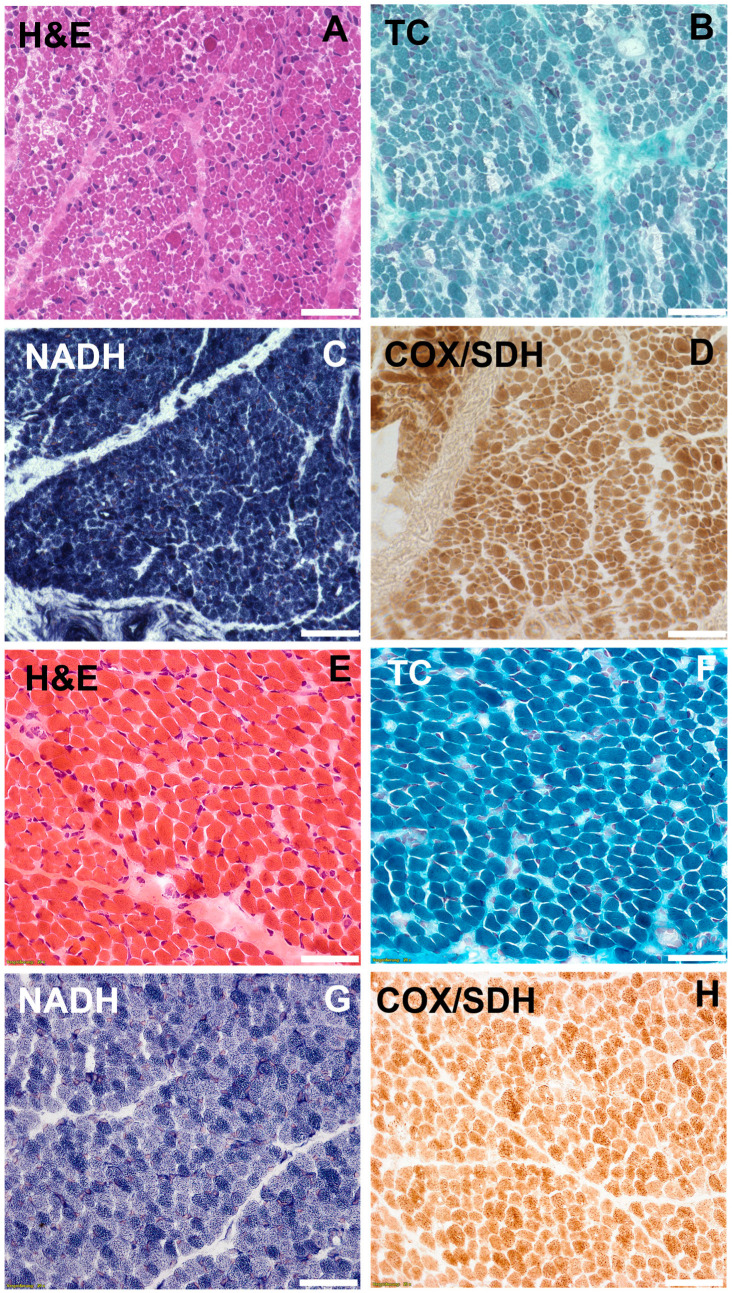
(**A**–**D**). Histochemical staining of vastus lateralis muscle in the index patient performed 10 days after birth showed pathological alterations including enhanced presence of peri- and endomysial fat cells, vacuole alterations, fiber size variations, and few internal myonuclei in H&E and Trichrome (TC) staining. NADH and COX/SDH staining revealed enhanced central agglomeration of NADH and SDH in the muscle fibers. (**E**–**H**). Stainings of a normal control muscle biopsy performed 10 days after birth (scale bar 50 µm).

A muscle biopsy of the vastus lateralis muscle was performed 10 days after birth which showed pathological alterations including fiber size variations, enhanced presence of peri- and endomysial fat cells, vacuole alterations, and few internal myonuclei in hematoxylin and eosin (H&E) stain (Figure 2). The muscle fiber diameter of type-1 fibers ranged from 1–24 μm. For type-2 fibers the diameter ranged from 1 to 17 µm (ref.: 9–21 µm in healthy children). Nicotinamide adenine dinucleotide (NADH) as well as cyclooxy-genase/succinate dehydrogenase (COX/SDH) staining revealed enhanced central agglomeration of NADH and SDH in the muscle fibers. Cores or minicores pathognomonic of *RYR1* mutations could not be found. Periodic acid Schiff (PAS) staining showed PAS-positive deposits. Electron microscopy of the muscle tissue revealed subsarcolemmal and intermyofibrillar accumulation of glycogen in the sarcoplasm and autophagic vacuoles.

### 2.2. Molecular Genetic Diagnostics

A panel analysis for myopathies including 128 genes was performed at the age of two months which revealed a known pathogenic splice variant in the *RYR1* gene in a heterozygous state: c.14364+1G>A (NM_000540.3). However, the healthy mother was shown to carry the same variant in a heterozygous state, suggesting that this variant alone could not be responsible for the patient’s severe phenotype. In order to identify a possible deletion or duplication on the second allele, a multiplex ligation-dependent probe amplification (MLPA) analysis of the gene was then performed, which was unremarkable. Analyses for myotonic dystrophy type 1 and spinal muscular atrophy also gave normal results. Therefore, it was concluded (2018) that the patient and his mother could either both be carriers of an autosomal recessive form of an *RYR1*-related myopathy which could not explain the patient’s symptoms or the patient could carry a second pathogenic *RYR1* variant on the paternal allele which had so far not been detectable with the routine methods used.

As part of the present study, we first performed WES in the patient which confirmed the previously described variant in the *RYR1* gene in a heterozygous state, but did not reveal a second pathogenic or likely pathogenic variant in this gene or any other myopathy-related gene. In the next step, the sample was analyzed using optical genome mapping (OGM), a method for detecting structural variation at high resolution [7] which had recently led to the detection of an X-chromosomal inversion in a patient with unsolved Duchenne muscular dystrophy in our center [8]. However, no pathogenic structural variant could be detected in our patient. Last, WGS was performed using the NovaSeq 6000 Sequencing System (Illumina, San Diego, CA, USA). In addition to the previously described variant, a second intronic variant was detected in the *RYR1* gene: c.10441−48G>A (NM_000540.3). This variant has already been described once in the literature by Shillington et al. (2021) in a severely affected child in a compound heterozygous state [9]. It was classified as likely pathogenic based on the RNA sequencing results presented in this study, confirming a splice defect [9]. Since RNA analyses demonstrating an effect on splicing had already been published for this variant and we did not have sufficient muscle biopsy material from our patient, we did not perform additional RNA analyses ourselves. Sanger sequencing revealed that the healthy father is heterozygous carrier of the deep intronic variant and, thus, confirmed the compound heterozygous state of the two variants in our patient.

### 2.3. Phenotypic Presentation in Comparison to Other Compound Heterozygous Cases

Given the highly variable phenotypes and the lack of reliable genotype–phenotype correlations for *RYR1* variants, we aimed to obtain a better understanding of the clinical impact of the new intronic splice variant. Therefore, we compared the phenotype observed in our patient to that of the patient from the literature, as well as five additional patients with other compound heterozygous *RYR1* variants that had been detected through routine WES in our center over the last 5 years (Table 1 and Table 2). The patient from Shillington et al. carried two splice variants, one at a canonical splice site and the abovementioned deeply intronic variant (position 48). Both variants were shown to affect splicing through RNA sequencing and therefore regarded as likely pathogenic [9]. The other five patients from our center each carried one pathogenic or likely pathogenic variant together with a second variant, so far classified as variants of unknown significance. However, taking into account the clinical phenotypes, the compound heterozygous state, and data from either muscle biopsy or MRI, they were interdisciplinarily interpreted as very likely causative (Table 1).

Comparison of the clinical data revealed that our index patient and the patient from Shillington et al., both carrying the novel splice variant together with a second pathogenic splice variant, showed a very severe phenotype and required constant ventilation from birth. However, the patient from Shillington et al. additionally showed a congenital heart defect (transposition of the great arteries), suffered from an intracerebral bleeding, and died in early infancy. In contrast, our index patient is non-ambulatory but alive at 6 years of age and can move a few fingers. Including the other five patients in the comparison of phenotypes revealed congenital onset of symptoms, predominant muscular hypotonia, and normal to slightly elevated CK levels as the main overlapping features, while overall clinical expression varied substantially. All but one of the five patients achieved free walking, but one lost ambulation again at the age of 20 years. Reduction in nerve conduction velocities was not evident for any of our patients but has been described for the patient from Shillington et al. The current clinical picture in our small cohort ranges from severe myopathy, wheelchair-bound and with constant ventilation (our index patient = patient 1), over slowly progressive myopathy leading to loss of ambulation in early adulthood, to mild non-progressive hypotonia without many restrictions in daily life. The family history for neuromuscular diseases was negative in all but one family: here, the index patient was the 42-year-old mother, whose sister carried the same two *RYR1* variants in a compound heterozygous state and showed similar muscular symptoms. Interestingly, two of her children inherited the known pathogenic *RYR1* variant and developed milder muscular symptoms, while the third child inherited the missense variant of unknown significance and showed a normal motor development.

**Figure 3:**
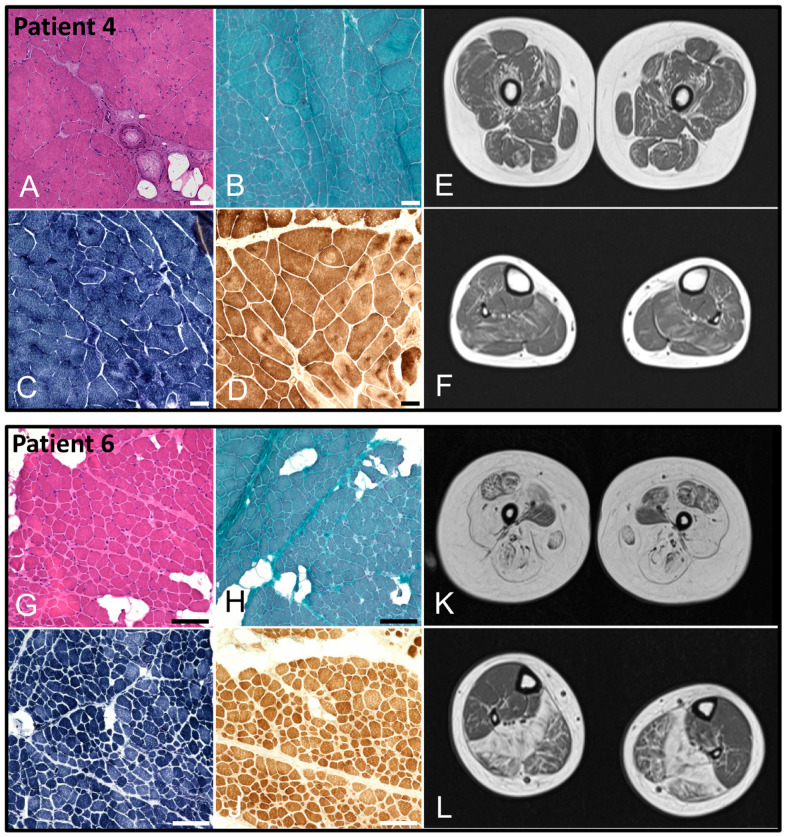
(**A**–**D**). Muscle biopsy of vastus lateralis muscle from patient 4 at the age of 9 years. Histochemical staining showed pathological alterations including presence of peri- and endomysial fat cells, fiber size variations with groups of atrophic fibers, internal myonuclei, and fiber splitting in H&E (**A**) and TC (**B**) staining. NADH (**C**) and COX/SDH (**D**) staining revealed enhanced central agglomeration of NADH and SDH in the muscle fibers building spoke-wheel–like structures (scalebar: 100 µm; control: Supplementary Figure S1). (**E**,**F**). MRI of the thigh and calves of patient 4 at the age of 9 years showing moderate, symmetric, reticular/streaky fat accumulation of lower limb muscles with involvement of quadriceps, biceps femoris, semitendinosus, semimembranosus, and soleus muscles and relative sparing of rectus femoris, gracilis, and tibialis posterior muscles. (**G**–**J**). Muscle biopsy of vastus lateralis muscle from patient 6 at the age of 1.5 years. Histochemical staining showed pathological alterations including presence of peri- and endomysial fat cells, fiber size variations, and fiber splitting in H&E (**G**) and TC (**H**) staining. Multiminicore-like alterations could be shown in NADH (**I**) and COX/SDH (**J**) staining (scalebar: 50 µm; control: Supplementary Figure S1). (**K**,**L**). MRI of the thigh and calves of patient 6 at the age of 13 years showing progressive, symmetric fat accumulation and atrophy of lower limb muscles with involvement of quadriceps, biceps femoris, semitendinosus, semimembranosus, and soleus muscles and relative sparing of adductor magnus, rectus femoris, gracilis, and tibialis posterior muscles.

Muscle biopsies were available only for patients 4 and 6. For patient 4, we found pathological alterations with enhanced central agglomeration of NADH and SDH, building spoke-wheel-like structures in the muscle fibers. Cores or minicores pathognomonic of *RYR1* mutations could not be detected (Figure 3A–D). In patient 6, besides pathological alterations in H&E and TC staining, we could find multiminicore-like alterations in NADH and COX/SDH staining (Figure 3G–J). For these two patients, MRI scans of the thigh and calf muscles were also available (Figure 3E,F,K,L, resp.). These showed symmetric fat accumulation and atrophy with a pattern consistent with typical MRI features described for *RYR1*-related myopathies [10]. For our index patient, however, an MRI was not performed, since the risk of the required anesthesia was considered too high.

**Table 1:** Compound heterozygous *RYR1* variants detected in 6 patients in our center and in the case report by Shillington et al., 2021 [9].

Patient	*RYR1* Variants	Variation Type	ACMG Classification	Previous Report of Variant
1	c.14364+1G>A, p.(?)	Splice	Pathogenic	[11,12,13]
	c.10441−48G>A, p.(?)	Splice	Likely pathogenic	[9]
2 ^+^	c.6274+1G>A, p.(?)	Splice	Likely pathogenic	[9]
	c.10441−48G>A, p.(?)	Splice	Likely pathogenic	[9]
3	c.2505del, p.(Pro836Leufs*48)	Frameshift	Pathogenic	[14,15,16,17]
	c.6197T>C, p.(Leu2066Pro)	Missense	Uncertain	-
4	c.1951C>T, p.(Arg651*)	Nonsense	Pathogenic	-
	c.11999_12001delATG, p.(Met4000del)	In-frame deletion	Uncertain	-
5	c.1951C>T, p.(Arg651*)	Nonsense	Pathogenic	-
	c.10883_10884delinsAA, p.(Arg3628Gln)	Missense/small indel	Uncertain	-
6	c.1250T>C, p.(Leu417Pro)	Missense	Likely pathogenic	[4,18,19]
	c.13677C>G, p.(Asn4559Lys)	Missense	Uncertain	-
7	c.5470C>T, p.Gln1824*	Nonsense	Pathogenic	-
	c.12138G>A, p.(Met4046Ile)	Missense	Uncertain	-

^+^ Patient 2 is not part of our center, he was described in Shillington et al., 2021 [9] and is used for comparison with our index patient.

**Table 2:** Comparison of phenotypic features in 6 patients with compound heterozygous *RYR1* variants detected in our center and in the patient from the case report by Shilling et al., 2021 ([9], P2).

	P1	P2 *	P3	P4	P5	P6	P7	%
**General information**								
Current age (yrs)	6	Death in infancy	28	14	9	14	42	n.a.
Gender	m	m	f	m	m	m	f	n.a.
Age of onset (months)	0	0	0	0	0	0	0	100%
Family history for myopathy	Negative	Negative	Negative	Negative	Negative	Negative	Positive ^€^	14%
**Neuromuscular symptoms**								
Ventilation after birth required	+	+	+	+	-	-	-	57%
Constant ventilation required	+	+	-	-	-	-	-	29%
Feeding difficulties	+	+	+	-	+	-	-	50%
Hypotonic facies	+	n.a.	+	-	+	-	-	50%
Elevation of CK levels	-	-	-	-	-	-	-	0%
Reduced endurance	n.a.	n.a.	-	+	+	-	+	60%
Hypermobile joints	+	n.a.	+	-	-	+	-	50%
Contractures	+	n.a.	+	-	-	+	+	67%
Dysarthria	+	n.a.	+	-	+	-	-	50%
Malignant hyperthermia	-	-	-	-	-	-	+(2 yrs)	14%
Reduced nerve conduction vel.	-	+	-	-	n.a.	-	-	17%
**Muscular hypotonia**								
Upper extremities affected	+	+	+	-	+	+	+	86%
Lower extremities affected	+	+	+	+	+	+	+	100%
Lower vs. upper extremities	=	=	>	>	>	>>	=	n.a.
**Motor milestones**								
Age at free walking	-	n.a.	18 months	18 months	18 months	(only few steps)	21 months	n.a.
Loss of ambulation	n.a.	n.a.	20 yrs	-	-	8 yrs	-	40%
**MRI pattern ^§^**								
AM > AD	n.a.	n.a.	n.a.	+	n.a.	+	n.a.	100%
V > RF	n.a.	n.a.	n.a.	+	n.a.	+	n.a.	100%
S > G	n.a.	n.a.	n.a.	-	n.a.	+	n.a.	50%
SO > GM	n.a.	n.a.	n.a.	+	n.a.	+	n.a.	100%
PG > TA	n.a.	n.a.	n.a.	-	n.a.	+	n.a.	50%
**Muscle biopsy**	Fiber size variation, few internal nuclei (Figure 2)	Fiber size variation, numerous immature type 2C muscle fibers	Fiber size variation	Fiber size variation, centronuclear myopathy (Figure 3)	-	Fiber size variation, chronic active myopathy, reduced α-dystroglycan (Figure 3)	Fibrolipomatosis, internal nuclei	
**Pregnancy issues**	Gest. diabetes, preeclampsia; birth at 34 + 6 w	-	Polyhydramnion, reduced fetal movements	Birth at 32 weeks	Reduced fetal movements	Polyhydramnion	-	
**Additional features**								
Heart diseases	-	Transposition of great arteries	Preexcitation syndrome	-	-	-	-	
Lung diseases	Ventilation required since birth	Ventilation required since birth	Progressive restrictive ventilation disease	Ventilation required for 2 weeks after birth	-	-	-	
Orthopedic diseases	Contractures	n.a.	Scoliosis	Genu valgus	Pes planus valgus	Cong. bilateral hip dysplasia	-	
Neurological diseases	Secondary microcephaly	Intracerebral hemorrhages, motor axonal polyneuropathy	-	-	-	-	Progressive hearing loss	
Congenital malformations	-	Transposition of great arteries	-	-	-	Congenital hip and knee contractures	Cleft lip/palate	
Cognitive impairment	-	n.a.	-	-	-	-	-	

* Patient from Shillington et al., 2021 [9]. ^€^ Sister compound heterozygous for the same *RYR1* variants; 2 children with one pathogenic variant and milder symptoms. § Adapted from Lawal et al., 2018 [10]; AM: M. adductus magnus, AL: M. adductus longus, V: M. vastus; RF: M. rectus femoris, S: M. sartorius, G: M. gracilis, SO: M. soleus, GM: M. gastrocnemius medialis, PG: Peroneus group, TA: M. tibialis anterior.

## 3. Discussion

Currently, routine genetic analyses for neuromuscular disorders include mainly MLPA or repeat expansion analyses for some specific phenotypes (e.g., spinal muscular atrophy and myotonic dystrophy, resp.) and WES. WES can (theoretically) cover all coding variation and the intron/exon boundaries, altogether approximately 2% of the whole human genome, but leaves out non-coding, potentially regulatory sequences as well as large structural variation. Therefore, it is an ongoing discussion whether WGS should be introduced as a first-line test into routine diagnostics [20]. In a retrospective assessment of 247 patients with unsolved neuromuscular disorders, the incorporation of genome sequencing, RNA sequencing, and protein investigations after WES increased the diagnostic yield from 40% to >60% [21].

We here describe a patient with a severe congenital myopathy who was known to carry one recessive pathogenic variant in *RYR1* from the first year of life. The second, likely pathogenic, *RYR1* variant was detected via WGS at the age of 6 years, further underlining the importance of WGS as compared to WES. However, WGS is limited at the moment by the fact that the clinical as well as the functional relevance is still completely unknown for a large amount of non-coding variation. WGS will render approximately 4,000,000–5,000,000 single-nucleotide variants (SNVs), 700,000–800,000 insertion–deletion variants, and 23,000–28,000 structural variants per genome as compared to the reference genome [22], and information in databases is still very limited for non-coding variants. In our case, the intronic variant at position −48 could only be classified as likely pathogenic because it had recently been described in another patient in the literature, and RNA analyses had already been performed to support a splicing defect; otherwise, it would have been filtered out by our bioinformatic pipeline. Therefore, a combination of WGS and transcriptome analyses may be helpful to detect more intronic splice variants in the future. Additionally, as for WES, information on variants discovered through WGS in international databases will certainly improve over the coming years, so using this approach as a first-line test will become more feasible.

*RYR1* variants account for a large proportion of cases with congenital myopathy, however, there is a very high degree of both clinical and genetic heterogeneity. Cohort studies have suggested some evidence for genotype–phenotype correlations [1,23], although the whole picture is far from being understood. Variants causing MH have been shown to be almost exclusively missense variants that occur more often in, but are not limited to, three hot spot regions [24]. On the other hand, autosomal recessive variants were found more randomly distributed throughout the gene [1]. Generally, autosomal recessive inheritance has been associated with more severe phenotypes, although some cases of early severe disease have also been described for dominant inheritance [25]. Further, within the recessive group, the presence of at least one hypomorphic (e.g., nonsense, frameshift, or splice site) variant has been linked to a more severe disease course as compared to two non-hypomorphic alleles [23]. However, in the largest studies published so far, there was either no patient with two hypomorphic variants [1] or no further distinction was made between patients carrying one or two hypomorphic alleles, most likely due to the small sample sizes [23].

Interestingly, both our patient and the patient described by Shillington et al. carry a combination of two different splice (and, thus, presumably hypomorphic) variants and show a phenotype at the severe end of the spectrum, with continuous ventilation required from birth. Similarly, Zecevic et al. recently described a family with two children showing fetal akinesia and early death who also carried two compound heterozygous *RYR1* splice variants [2]. On the other hand, there are some significant differences between our patient and the patient from Shillington et al. Additional to his severe myopathy, the patient from the literature also showed a conotruncal heart defect and an intracerebral bleeding. Both probably contributed to the very severe phenotype, eventually leading to the decision to discontinue the ventilation, and the patient passed away in early infancy. The authors discuss that both the heart defect and the intracerebral bleeding could be independent from the *RYR1*-related myopathy, but may also constitute very rare manifestations of the *RYR1*-associated spectrum [9]. Our index patient shows a severe cause of the myopathy, but neither he nor any of the additional patients exhibit signs of heart involvement or a bleeding disorder. These findings rather argue against this theory but, of course, cannot exclude it.

To get a better idea of the clinical spectrum associated with autosomal recessive *RYR1*-related myopathy, we included data of five additional patients from our center detected via exome sequencing over recent years. They each carry a known pathogenic variant (four of them hypomorphic, one missense) in compound heterozygous state with a so far unknown, but in our opinion likely causative, missense or in-frame (e.g., non-hypomorphic) variant. In comparison to our index patient, they all showed a substantially milder disease course, with walking freely achieved (at least for a while) for all but one of them. Congenital onset of symptoms, predominant muscular hypotonia, and normal to slightly elevated CK levels were the main overlapping features. However, the observed range of severity was very broad, with one patient only able to move a few steps, another with loss of ambulation at 20 years of age, and two children with a rather mild disease course without many restrictions in daily life (although they are still too young to assess the later disease course). These observations are generally compatible with a very high clinical heterogeneity in *RYR1*-related myopathies, as described by many authors [4]. We underline the suggestion that two hypomorphic *RYR1* variants, in this case two splice variants, seem to be associated with a more severe disease course and severe congenital myopathy. However, we are fully aware that the group of patients is far too small to conclude any valid genotype–phenotype correlations. So far, predicting the phenotype based on genotype data, for example, in a prenatal setting, is only very limited. More widespread use of genome sequencing will hopefully lead to better genotype–phenotype correlations, that could help to guide and counsel patients and families in the future.

The muscle biopsy of our index patient showed pathological alterations including enhanced central agglomeration of NADH and SDH in the muscle fibers, compatible with *RYR1*-related myopathy, but no typical cores or multiminicores [26,27]. However, it has been clearly observed over the years that these histopathological features are non-specific and may change over time. The muscle tissue was taken at a very early stage (10 days of life), therefore we cannot exclude that a biopsy at a later age may have shown more pathognomonic features. In the muscle tissue of the patient described by Shillington et al., staining with a RYR1-specific antibody was significantly reduced compared to control tissue. Unfortunately, there was not enough muscle tissue left over from our patient to perform this analysis.

In conclusion, we describe a second patient in whom WGS led to the definitive diagnosis of an *RYR1*-related congenital myopathy due to a deep intronic splice variant in this gene. Elucidating the exact genetic cause of the muscular disorder for each patient has become more and more important over the past years, since individualized and mutation-specific therapeutic approaches are rapidly evolving [28,29]. For example, for spinal muscular atrophy, both antisense-oligonucleotide-based treatments as well as gene therapy are now routinely available and have led to substantially improved outcomes [30]. For many other neuromuscular diseases, similar advances are evaluated in preclinical or clinical studies. RYR1-related myopathies pose some difficulties for the development of such new techniques, due to the very large size of the gene and the presence of numerous variants that often occur only in a single family. Nevertheless, there are ongoing efforts to develop novel therapeutic options [31,32], hopefully leading to a better individualized treatment in the near future.

## 4. Materials and Methods

### 4.1. Whole Exome Sequencing and Segregation Analysis

WES libraries were constructed using the Twist Comprehensive Exome/Twist Library Preparation Kit (V2) (Twist Bioscience, South San Francisco, CA, USA) and sequenced as 2 × 150 nt paired end reads on a NextSeq1000 instrument (Illumina, San Diego, CA, USA). Sequence alignment, variant calling, and annotation were performed using the Varvis bioinformatics pipeline v1.22 (Limbus Medical Technologies GmbH, Rostock, Germany). Detected variants were evaluated based on the ACMG classification system [33] and the patient’s phenotype. Segregation analysis was performed with Sanger sequencing (information on primers available upon request).

### 4.2. Optical Genome Mapping

High-molecular-weight (HMW) DNA was isolated from frozen EDTA blood using the Bionano SP Blood and Cell Culture DNA Isolation Kit v2 according to the manufacturer’s instructions (Bionano Genomics, San Diego, CA, USA). Specific sequences of the DNA sample were labeled using the Direct Label and Stain kit. The labeled HMW DNA was loaded on a Bionano Genomics Saphyr Chip G2.3. Linearization and imaging were performed on a Saphyr instrument (Bionano Genomics, San Diego, CA, USA). Bioinformatic processing and analysis were performed using Bionano Solve 3.7.2 and Bionano Access 1.7.2 software. The de novo assembly pipeline was used to detect hereditary and de novo variants in the patient’s genome. The identified structural variants were filtered using the default filter settings. We examined structural variants that were present in 1% or less of the control datasets in the manufacturer’s control population which includes de novo assembly DLE-1 datasets from 179 ethnically diverse individuals.

### 4.3. Whole Genome Sequencing

The concentration of DNA was measured using the Qubit Fluorometric Quantitation and the DNA Broad-range Assay (Thermo Fisher Scientific, Waltham, MA, USA). For the library preparation, 350 ng of genomic DNA was fragmented to ~450 bp pairs using the DNA PCR-Free Prep, Tagmentation (Illumina, San Diego, CA, USA). The resulting libraries typically presented a concentration of 1.5–3 ng/µL and were sequenced as paired end 150 bp reads on an Illumina NovaSeq6000 (Illumina, San Diego, CA, USA) with a sequencing depth of approximately 120 Gb. Quality control of the generated fastq files, as well as read mapping, variant calling, and annotation, was performed using the megSAP pipeline (https://github.com/imgag/megSAP; accessed on 24 August 2023) and GRCh38 as a reference genome. Variants were viewed and prioritized using GSvar (https://github.com/imgag/ngs-bits/tree/master/doc/GSvar; accessed on 24 August 2023).

### 4.4. Muscle Biopsy

Hematoxylin-and-eosin (H&E) staining was performed following standard procedures [34]. First, unfixed serial skeletal muscle cryosections of 10 µm in thickness were incubated with hematoxylin for 30 s; after this, washing steps and dehydration steps with ethanol followed. Then, sections were incubated with eosin for 10 s and washed in several steps with ethanol and xylol. For NADH staining, muscle tissue was incubated with NADH solution containing 10 mg of nitro blue tetrazolium chloride, 8 mg of NADH, and 10 mL of 0.2 M phosphate buffer for 30 min at 37 °C. After washing with distilled water and treatment with solutions of increasing alcohol levels, the tissue was incubated with xylol. For COX/SDH, muscle tissue was incubated with COX solution containing 10 mg of diaminobenzidine, 10 mL of 0.05 M phosphate buffer, 750 mg saccharose, and 20 mg of cytochrome C for 2 h at 37 °C. After washing steps with distilled water, cover slips were incubated with SDH solution containing 100 µL of 130 mM natrium-succinate, 100 µL of 2 mM phenazine methosulfate, 10 µL of 100 mM sodium azide, and 800 µL of 1875 mM nitro blue tetrazolium chloride for 1 h at 37 °C. After washing with distilled water and treatment with solutions of increasing alcohol levels, the tissue was incubated with xylol. Immunofluorescence images were recorded using an Olympus microscope (Olympus IX83, Olympus, Hamburg, Germany) with connected cameras (XM10 for immunofluorescence and XC50 for H&E; Olympus) and CellSens software (CellSens Dimension version 1.17) for data processing. Histological images were recorded using a Keyence BZ-X810 microscope and BZ Series Application version 01.03.00.01 (Keyence, Osaka, Japan).

## Data Availability

The data presented in this study are available upon request from the corresponding author.

## Supplementary Materials

The following supporting information can be downloaded at: https://www.mdpi.com/article/10.3390/ijms251910867/s1.
