# Supplementary material for: Compound Heterozygous RYR1 Variants in a Patient with Severe Congenital Myopathy: Case Report and Comparison with Additional Cases of Recessive RYR1-Related Myopathy
Source: Int J Mol Sci. 2024 Oct 9;25(19):10867. doi: 10.3390/ijms251910867 (PMC11477233; doi:10.3390/ijms251910867)
Supplement: Supplementary file 1 [file ijms-25-10867-s001.zip › ijms-3160941-supplementary.pdf]

Supplementary figure S1

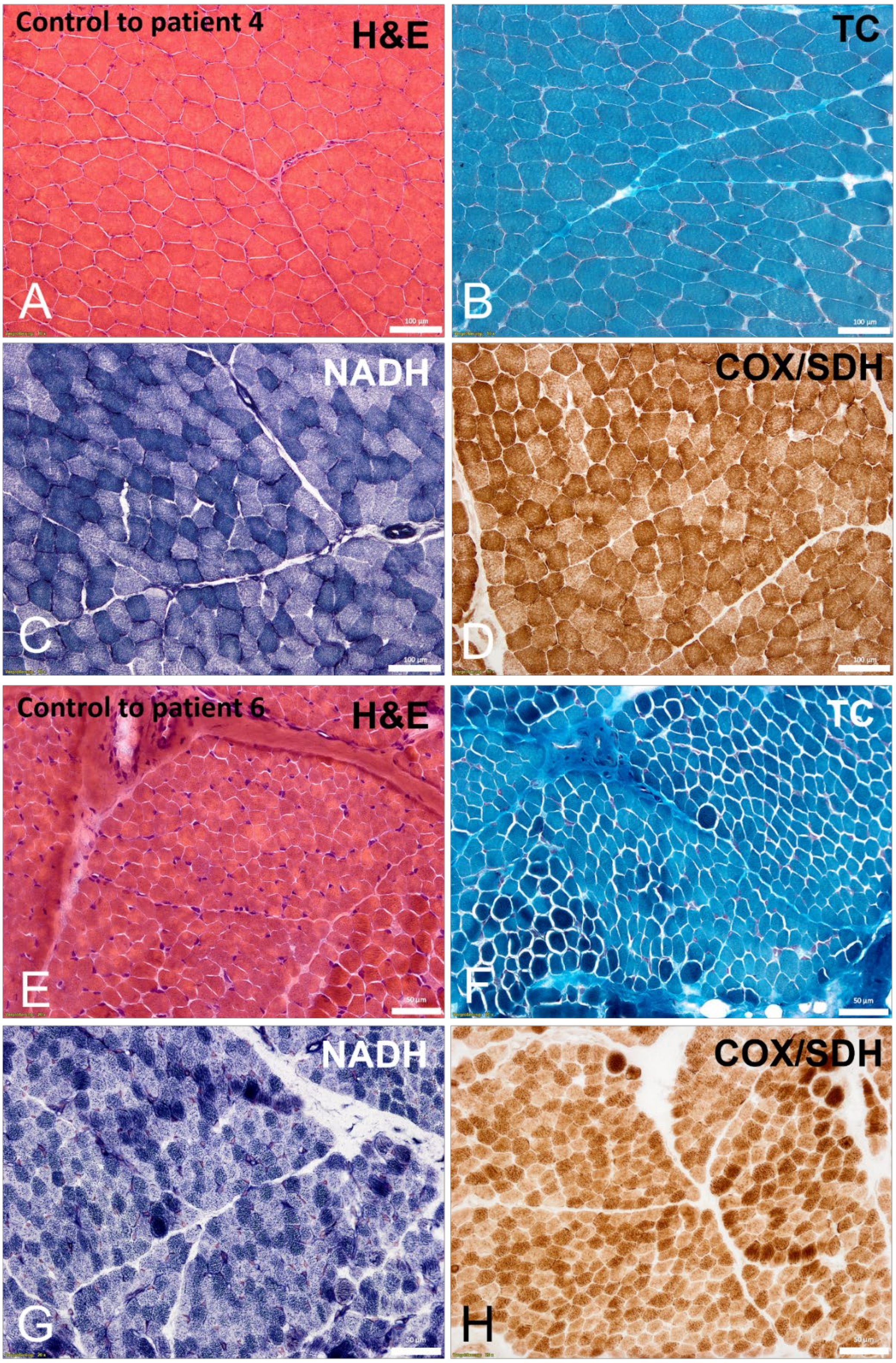

A-D: Control sample for muscle biopsy of patient 4. Histochemical stainings of a control muscle biopsy performed at the age of 8.6 years (scalebar 100 μm)

E-H: Control sample for muscle biopsy of patient 6. Histochemical stainings of a control muscle biopsy performed at the age of 1.2 years (scalebar 50 μm)
